# TAK1 regulates Paneth cell integrity partly through blocking necroptosis

**DOI:** 10.1038/cddis.2016.98

**Published:** 2016-04-14

**Authors:** A N Simmons, R Kajino-Sakamoto, J Ninomiya-Tsuji

**Affiliations:** 1 Department of Biological Sciences, North Carolina State University, Raleigh, NC 27695-7633, USA

## Abstract

Paneth cells reside at the base of crypts of the small intestine and secrete antimicrobial factors to control gut microbiota. Paneth cell loss is observed in the chronically inflamed intestine, which is often associated with increased reactive oxygen species (ROS). However, the relationship between Paneth cell loss and ROS is not yet clear. Intestinal epithelial-specific deletion of a protein kinase *Tak1* depletes Paneth cells and highly upregulates ROS in the mouse model. We found that depletion of gut bacteria or myeloid differentiation factor 88 (*Myd88*), a mediator of bacteria-derived cell signaling, reduced ROS but did not block Paneth cell loss, suggesting that gut bacteria are the cause of ROS accumulation but bacteria-induced ROS are not the cause of Paneth cell loss. In contrast, deletion of the necroptotic cell death signaling intermediate, receptor-interacting protein kinase 3 (*Ripk3*), partially blocked Paneth cell loss. Thus, *Tak1* deletion causes Paneth cell loss in part through necroptotic cell death. These results suggest that TAK1 participates in intestinal integrity through separately modulating bacteria-derived ROS and RIPK3-dependent Paneth cell loss.

TAK1 (MAP3K7) is a member of mitogen-activated protein kinase kinase kinase (MAP3K), and an indispensable signaling intermediate of proinflammatory cytokine and Toll-like receptor (TLR)/NOD-like receptor signaling pathways leading to activation of transcription factors, NF-*κ*B and AP-1 (reviewed by Mihaly *et al.*^1^). NF-*κ*B and AP-1 induce expression of a number of proinflammatory and cell survival genes including several antioxidant genes.^2^ TAK1 was also found to regulate a redox transcription factor, nuclear factor erythroid 2 (NF-E2)-related factor 2 (Nrf2).^3^ The levels of Nrf2 protein and its target gene expression were downregulated in *Tak1*-deficient tissue culture cells and intestinal epithelium.^3^ Thus, through these transcription factors, TAK1 participates in the maintenance of the cellular antioxidant system. Deletion of *Tak1* impairs the cellular redox balance resulting in reactive oxygen species (ROS) accumulation in cultured cells.^4, 5, 6^ *Tak1* deficiency causes cell death primarily through apoptosis,^7^ but also induces a regulated type of necrosis so-called necroptosis.^8, 9, 10, 11^ Increased ROS are causally associated with apoptosis in *Tak1*-deficient cells,^4, 5, 12^ whereas the mechanism by which *Tak1* deficiency induces necroptosis is not yet clear.

In a mouse model, intestinal epithelial-specific *Tak1* deletion causes cell death, severe inflammatory conditions and perinatal animal lethality.^13^ Ablation of the proinflammatory cytokine TNF by tumor necrosis factor 1 receptor 1 (*Tnfr1*) gene deletion effectively alleviates inflammation. Adult mice harboring intestinal epithelial-specific *Tak1* deletion on *Tnfr1*^*−/−*^ background do not show observable health problems.^14^ However, the *Tak1*-deficient intestine still exhibits increased apoptosis in the crypt of the ileum and milder inflammatory conditions, which are similar to human ileitis.^3^ ROS are highly increased in the *Tak1*-deficient intestinal epithelium even on a *Tnfr1*^*−/−*^ background.^3^ Furthermore, we found that almost no Paneth cells were observed in the *Tak1*-deficient small intestine (shown in the current study). Paneth cells reside at the base of the crypts in the small intestine and are specialized to secret antimicrobial enzymes and peptides such as lysozyme C and defensins, which control commensal microbiota.^15^ Paneth cells are a unique cell type among the specialized intestinal epithelial cells, which have a very long life span of around 6–8 weeks, while other cells are constantly renewed about every 3–6 days in the mouse intestinal epithelium.^16, 17^ Inflammatory bowel disease (IBD) is a group of chronic inflammatory diseases of the intestine, which is characterized by increased ROS in the intestinal epithelium and is sometimes associated with degradation of Paneth cells.^18, 19^ One type of IBD, Crohn's disease, is specifically characterized by ileitis and dysfunction of Paneth cells, which resemble the *Tak1*-deficient intestinal epithelium. In the current study, we sought to determine the mechanism by which *Tak1* deficiency causes IBD-like pathology, that is, increased ROS and loss of Paneth cells. We postulated two scenarios: one is that *Tak1* deficiency causes ROS accumulation because of an impaired cellular redox system, which is the cause of Paneth cell loss; the other is that *Tak1* deficiency causes Paneth cell death, which results in the disruption of normal gut microbiota leading to increased ROS. A better understanding of the relationship between two major IBD disorders: ROS and Paneth cell loss could shed new insights into IBD pathogenesis, which is still largely undetermined.

## Results

### Intestinal epithelial-specific deletion of *Tak1* depletes Paneth cells

**Figure 1:**
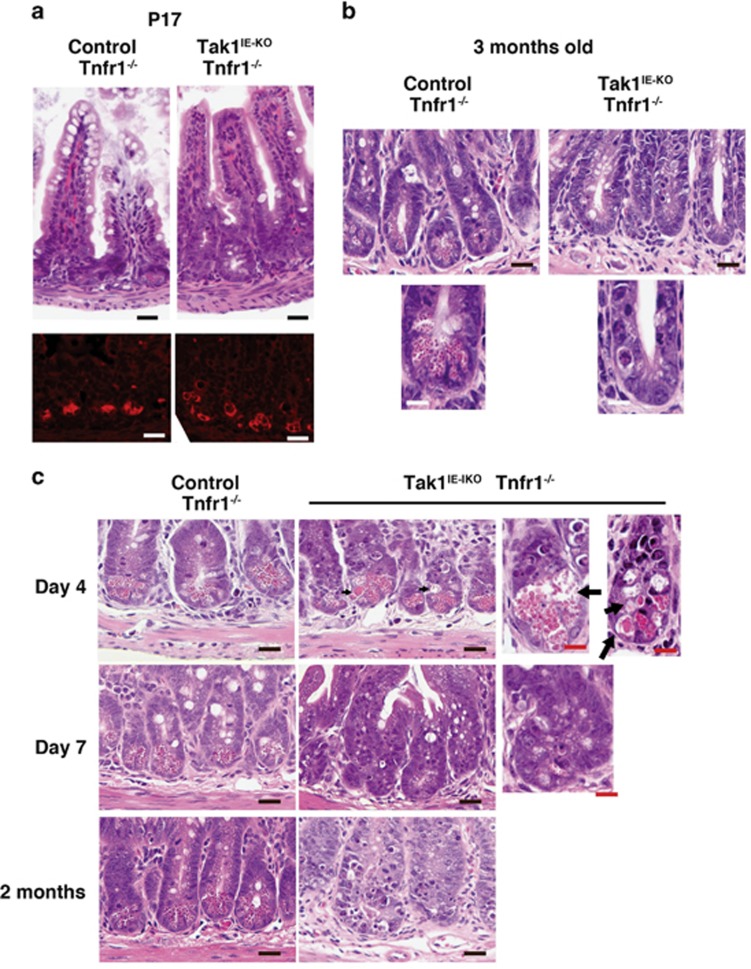
Tak1 deletion depletes Paneth cells. (**a**) H&E staining (upper panels) and immunofluorescence staining of Paneth cell marker, lysozyme (red) (lower panels) of control and the non-inducible version of intestinal epithelial-specific *Tak1-*deficient ileum on a *Tnfr1*^*−/−*^ background at postnatal day 17. Scale bars, 20 *μ*m. See also Supplementary Figures S1A–C. (**b**) H&E staining of the crypts of ileum (3 months old). Scale bars; upper panels, 20 *μ*m; lower panels (high magnifications), 10 *μ*m. (**c**) H&E staining of control and the inducible version of intestinal epithelial-specific *Tak1*-deficient crypts of ileum on a *Tnfr1*^*−/−*^ background. Tamoxifen was injected for three consecutive days and analyzed at 4, 7 or 2 months after the tamoxifen treatment. Black arrows indicate structurally disrupted Paneth cells. Black scale bars, 20 *μ*m: red scale bars, 10 *μ*m. See also Supplementary Figures S1D–E

To determine the mechanism by which *Tak1* deletion causes IBD-like intestinal injury, we initially re-evaluated the intestinal morphology in the *Tak1*-deficient intestinal epithelium. We used mice having intestinal epithelium-specific *Tak1* deletion on a *Tnfr1* null background (Tak1^IE-KO^ Tnfr1^*−/−*^). Although some but not all Tak1^IE-KO^ Tnfr1^*−/−*^ mice develop inflammatory conditions around postnatal day 15–17,^13^ once they reach the adult stage, Tak1^IE-KO^ Tnfr1^*−/−*^ mice do not show appreciable abnormalities.^14^ Intestinal epithelium with compound deletion of *Tak1* and *Tnfr1* exhibits only a mild increase of inflammatory cytokines, IL-1 and IL-6, and a chemokine, C-X-C motif ligand 2.^3^ However, *Tnfr1* deletion does not reduce the number of dying cells or the level of ROS in the *Tak1*-deficient intestinal epithelium.^3^ We previously reported that goblet and enteroendocrine cells are normally developed around birth and the numbers of those cells are not altered by *Tak1* deficiency at postnatal day 0 (P0).^13^ In wild-type mice, Paneth cells become detectable around 2–3 weeks of age concomitantly with the establishment of commensal microbiota.^20^ To detect Paneth cells, we performed immunofluorescence staining of lysozyme, which is selectively expressed in Paneth cells, and Alcian blue staining, which detects acidic mucins in goblet cells and granules in Paneth cells.^21^ At P17, as Paneth cells are not yet fully matured, we observed two or three lysozyme-positive cells and weak Alcian blue staining at the base of crypt in both no-Cre Tnfr1^*−/−*^ and Tak1^IE-KO^ Tnfr1^*−/−*^ (Figure 1a, bottom panels, Supplementary Figures S1A and 1B, and also see ref. 13). Thus, Paneth cells are developed even in *Tak1*-deficient intestinal epithelium. Architecture of the small intestine in Tak1^IE-KO^ Tnfr1^*−/−*^ mice was largely intact at P17 (Figure 1a, upper panels and also see ref. 13). The total number of intestinal epithelial cells per crypt did not decrease in Tak1^IE-KO^ Tnfr1^*−/−*^ mice (Figure 1a, upper panels and also see ref. 13). These indicate that *Tak1* deficiency does not impair intestinal epithelial stem cells or their ability to differentiate toward specialized intestinal epithelial cells including Paneth cells. However, we found that Paneth cells were completely depleted in the adult (3-month-old) Tak1^IE-KO^ Tnfr1^*−/−*^ mice (Figure 1b). Thus, Paneth cells can complete their differentiation processes in the *Tak1*-deficient intestinal epithelium but they are not maintained.

**Figure 2:**
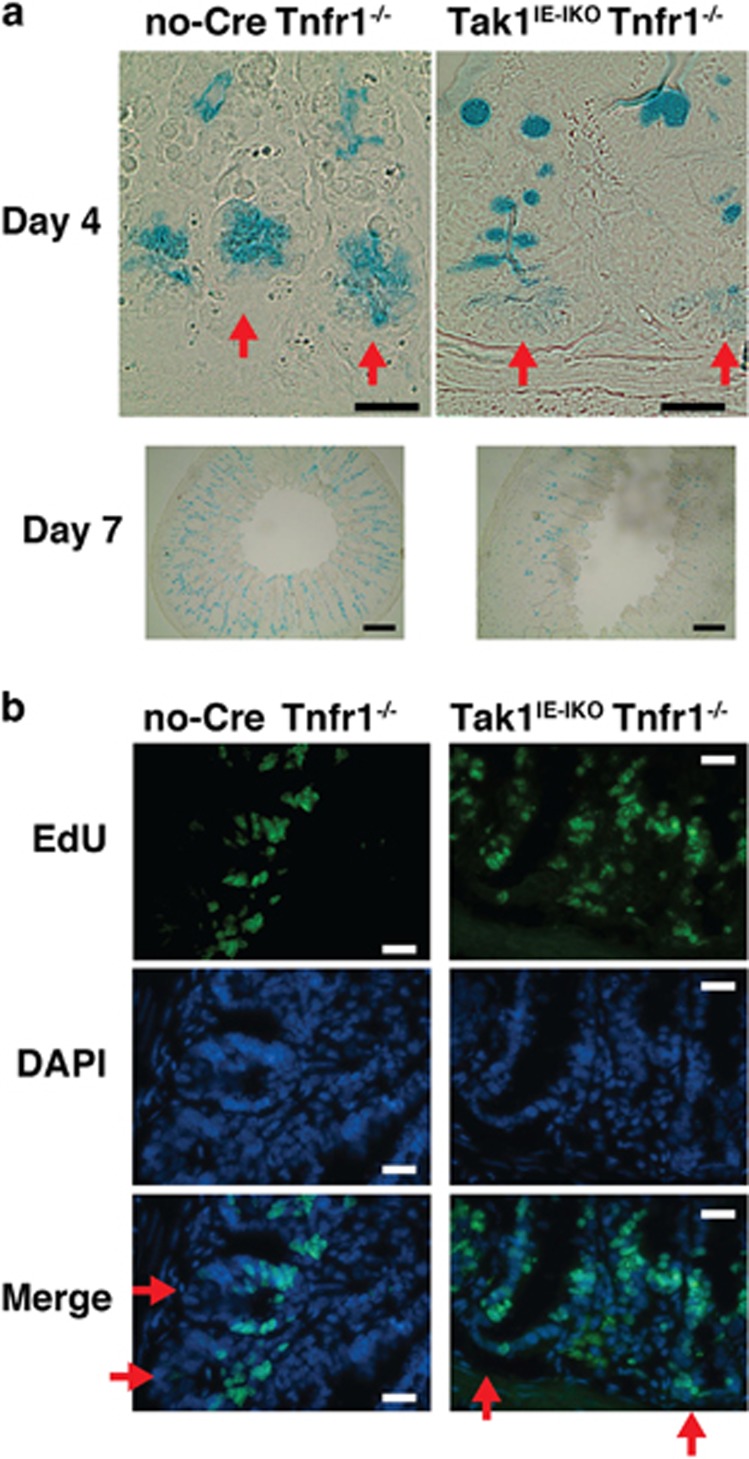
Tak1 deletion depletes Paneth cells without altering cell proliferation. (**a**) Alcian blue staining of no-Cre Tnfr1^*−/−*^ and Tak1^IE-IKO^ Tnfr1^*−/−*^ at day 3 (upper panels) or 7 (lower panels) after tamoxifen injection. To visualize weak staining of Alcian blue, no counterstaining was performed. Goblet cells were observed in lower magnification images (lower panels). Red arrows indicate the bottom of crypts. Scale bars, 50 *μ*m (upper panels); 200 *μ*m (lower panels). (**b**) No-Cre Tnfr1^*−/−*^ and Tak1^IE-IKO^ Tnfr1^*−/−*^ at day 14 after tamoxifen injection were treated with EdU for 4 h. EdU and DAPI staining are shown. Scale bars, 50 *μ*m. Red arrows indicate the bottom of crypts

To further investigate Paneth cell loss in the *Tak1*-deficient intestinal epithelium, we used mice carrying an inducible intestinal epithelial-specific *Tak1* gene deletion system on a *Tnfr1*^*−/−*^ background, *villin.CreER*^*T2*^ *Tak1*^*flox/flox*^ *Tnfr1*^*−/−*^ (Tak1^IE-IKO^ Tnfr1^*−/−*^). In this system, TAK1 is intact without an inducer of gene deletion, tamoxifen, and, upon intraperitoneal injection of tamoxifen for 3 consecutive days (day 3), intestinal epithelium TAK1 protein was diminished and *Tak1* deletion was afterward maintained without additional tamoxifen treatment (Supplementary Figure S1C). We found that Paneth cells (granulated cells in the base of crypts) were gradually decreased starting at day 4 after tamoxifen treatment and depleted around day 7 (Figure 1c). As heterozygous deletion of *Tak1*, *Villin.CreER*^*T2*^ *Tak1*^*flox/+*^ *Tnfr1*^*−/−*^, did not exhibit any abnormality with tamoxifen treatment (Supplementary Figure S1D), Paneth cell loss is dependent on *Tak1* deletion but not on artifacts from inducible Cre expression. Alcian blue staining at the base of crypts, was much weaker in Tak1^IE-IKO^ Tnfr1^*−/−*^ at day 4 after tamoxifen injection (Figure 2a; upper panels). Goblet cells (strong Alcian blue-positive cells) were decreased but still observable at day 7 after tamoxifen injection (Figure 2a; lower panels). Thus, both Paneth and goblet cells seem to be sensitive to *Tak1* deletion, but the impact of *Tak1* deletion is more profound in Paneth cells. Overall intestinal architecture (villi and crypts) was largely intact (see Figure 2a, bottom panels), but cell alignment in the Tak1^IE-IKO^ Tnfr1^*−/−*^ crypt was disorganized (Figure 1c). Whereas proliferating cells were similarly detected in both control and *Tak1*-deficient crypts, proliferating cells were occasionally found outside the normal transient-amplifying cell area such as in the base of crypt in Tak1^IE-IKO^ Tnfr1^*−/−*^ crypt (Figure 2b). We note here that, as *Tak1* deficiency mainly induces cell death within the area where proliferative cells reside as shown later, ectopic cell proliferation may be due to cell death-induced compensatory proliferation. In contrast to the small intestine, the colon was found to be relatively intact in Tak1^IE-IKO^ Tnfr1^*−/−*^ mice even after 2 months (Supplementary Figure S1E). Collectively, *Tak1* deficiency predominantly affects Paneth cell integrity and cell alignment in the small intestinal crypts.

Paneth cell loss was observed around day 7, which is much shorter than the lifespan of Paneth cells. Thus, the cause of Paneth cell depletion should not be due to impairment in the renewal processes but should be due to premature removal (cell death) of pre-existing Paneth cells. Indeed, we observed morphologically disrupted Paneth cells in Tak1^IE-IKO^ Tnfr1^*−/−*^ crypt at day 4 (Figure 1c, top right panel, arrows). These results suggest that *Tak1* deletion induces Paneth cell depletion, which is likely to be caused by Paneth cell death.

### Gut bacteria are the cause of accumulation of ROS in the *Tak1*-deficient intestinal epithelium

**Figure 3:**
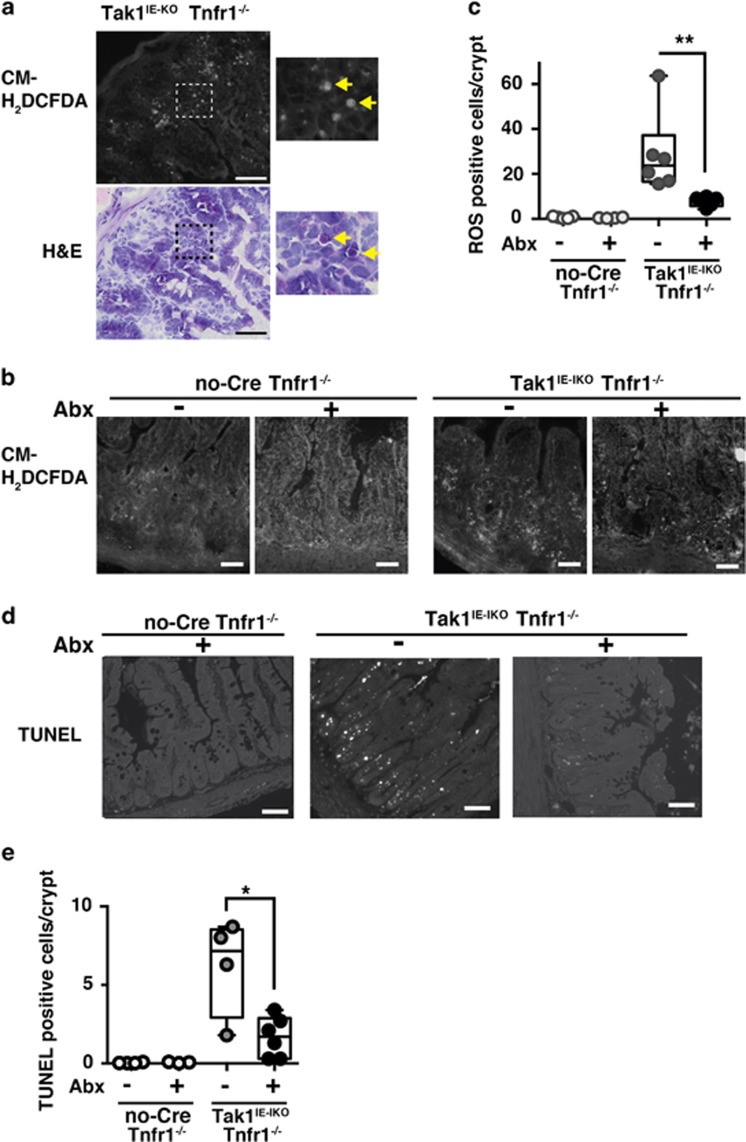
Antibiotic treatment reduces ROS and apoptosis. (**a**) ROS were determined by CM-H_2_DCFDA staining using freshly prepared unfixed cryosections of Tak1^IE-IKO^ Tnfr1^*−/−*^ small intestine at day 14 after tamoxifen injection (upper panels). The section was subsequently fixed and stained by H&E. The same position was photographed. Higher magnification (lower panel) or enlarged (upper panel) images of the dash line boxes in the right panels are shown in the left panels. Yellow arrows indicate the ROS-positive cells. Scale bars, 50 *μ*m. (**b**) Control no-Cre Tnfr1^*−/−*^ and Tak1^IE-IKO^ Tnfr1^*−/−*^ mice were treated with water alone or antibiotic cocktail water for 4 weeks and tamoxifen was injected once per day for three consecutive days. Days 7–10 after tamoxifen injection, fresh ileum sections were stained with CM-H_2_DCFDA. Scale bars, 40 *μ*m. (**c**) ROS-positive cells in each crypt of samples in (**b**) were counted, and the data shown are from four to six mice of the average of ROS-positive cells in more than 100 crypts per mouse. Control Tnfr1^*−/−*^ mice; without (*n*=5) and with (*n*=4) antibiotics (Abx). Tak1^IE-IKO^ Tnfr1^*−/−*^ mice; without (*n*=6) and with (*n*=6) antibiotics. All data points are shown in the box and whisker plots: median and distribution of 50% of values are shown in the box: whiskers indicate distribution of minimum and maximum values. **P<0.01 (one-way ANOVA). (**d**) TUNEL staining. Scale bars, 50 *μ*m. (**e**) TUNEL-positive cells were counted (more than 30 crypts per mouse). Control Tnfr1^*−/−*^ mice; without (*n*=4) and with (*n*=3) antibiotics (Abx). Tak1^IE-IKO^ Tnfr1^*−/−*^ mice; without (*n*=4) and with (*n*=6) antibiotics. All data points are shown in the box and whisker plots: median and distribution of 50% of values are shown in the box: whiskers indicate distribution of minimum and maximum values. *P<0.05 (one-way ANOVA)

Intestinal epithelial-specific *Tak1* deficiency induces ROS accumulation and cell death.^3^ We assessed ROS by using a general peroxide detection agent, CM-H_2_DCFDA, which is converted to a fluorescent product by cellular peroxides and is trapped inside of the cells.^22^ Earlier studies have shown that CM-H_2_DCFDA staining is capable of detecting ROS in tissue sections of the intestine^3^ and in the endothelium^23^ when fresh unfixed tissue sections are used. ROS-positive signals were validated by their disappearance when treated with the ROS scavenger, butylated hydroxyanisole as shown previously.^3^ We show that a number of cells were stained positive in Tak1^IE-IKO^ Tnfr1^*−/−*^ intestinal epithelium, whereas almost no cells were positive in *Tak1* intact controls (Figures 3a and b). CM-H_2_DCFDA staining-positive cells had unusual morphology compared with adjacent staining-negative intestinal epithelial cells (Figure 3a). Those are typically round and show condensed or dispersed nuclei, which are consistent with the histological features of apoptosis (Figure 3a) and clearly different from immune cells. CM-H_2_DCFDA staining-positive cells were found mainly in the lower part of the crypts (Figures 3a and b). This raises the possibility that ROS-induced apoptosis is the cause of Paneth cell loss. To test this, we first attempted to reduce ROS in the Tak1^IE-IKO^ Tnfr1^*−/−*^ intestinal epithelium. Bacterial moieties are major inducers of ROS in the intestinal epithelium (reviewed by Lambeth and Neish^24^). Thus, we postulated that depletion of gut bacteria could reduce ROS in the *Tak1*-deficient intestinal epithelium. We treated mice with an antibiotic cocktail, ampicillin (1 g/l), vancomycin (0.5 g/l), neomycin sulfate (1 g/l) and metronidazole (1 g/l), which is commonly used for depletion of commensal bacteria,^25^ for 4 weeks and subsequently treated with tamoxifen to delete *Tak1*. Bacteria were effectively reduced in this treatment (Supplementary Figure S2A). In the absence of antibiotic treatment, ROS were highly increased by *Tak1* gene deletion and ROS accumulation was predominantly observed in the lower part of the crypts at days 7–10 after tamoxifen treatment (Figure 3b), which is consistent with our previous results at day 3 after tamoxifen treatment.^3^ The level of ROS was greatly reduced with the pre-treatment of antibiotics (Figures 3b and c). Cell death was assessed by terminal deoxynucleotidyl transferase dUTP terminal nick-end labeling (TUNEL) staining, which were also observed predominantly in the lower part of the crypts (Figure 3d), and antibiotic treatment reduced the number of TUNEL-positive cells in the Tak1^IE-IKO^ Tnfr1^*−/−*^intestinal epithelium (Figures 3d and e). Cleaved caspase-3-positive cells were also increased in the Tak1^IE-IKO^ Tnfr1^*−/−*^ intestinal epithelium, which were reduced by antibiotic treatment (Supplementary Figures S2B and S2C). These results suggest that commensal bacteria are one of the major causes of ROS accumulation and cell death in the *Tak1*-deficient intestinal epithelium.

### TLR-MyD88 pathway mediates ROS accumulation

**Figure 4:**
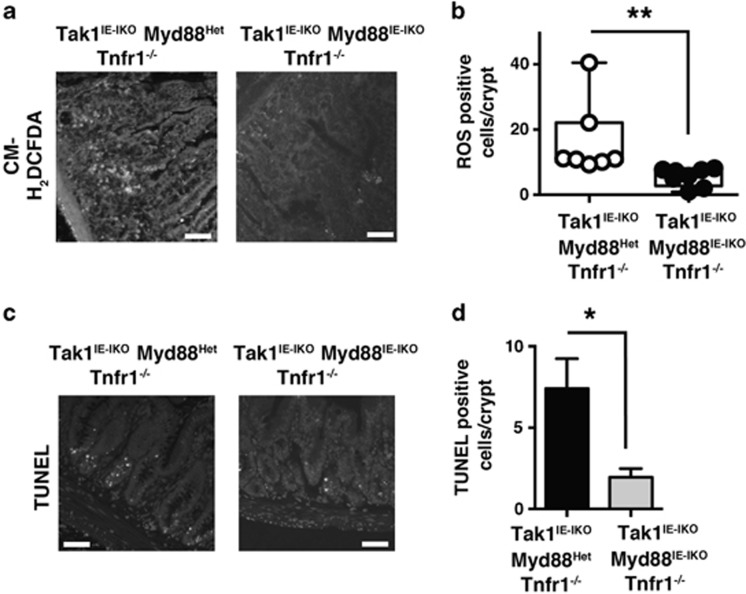
Myd88 deletion reduces ROS and apoptosis. (**a**) ROS-sensitive dye staining of *Myd88* heterozygous or *Myd88* homozygous deletion Tak1^IE-IKO^ Tnfr1^*−/−*^ ileum crypts at days 10–12 after tamoxifen injection. Scale bars, 50 *μ*m. (**b**) ROS-positive cells in each crypt of samples in (**a**) were counted (more than 100 crypts per mouse). *Myd88* heterozygous deletion Tak1^IE-IKO^ Tnfr1^*−/−*^ mice; *n*=7; *Myd88* homozygous deletion Tak1^IE-IKO^ Tnfr1^*−/−*^ mice; *n*=8. All data points are shown in the box and whisker plots: median and distribution of 50% of values are shown in the box: whiskers indicate distribution of minimum and maximum values. **P<0.01 (two-tailed unpaired Student's *t*-test). (**c**) TUNEL staining. Scale bars, 50 *μ*m. (**d**) Quantification of TUNEL-positive staining (more than 30 crypts per mouse). *Myd88* heterozygous deletion Tak1^IE-IKO^ Tnfr1^*−/−*^ mice; *n*=3; *Myd88* homozygous deletion Tak1^IE-IKO^ Tnfr1^*−/−*^ mice; *n*=3. Means±S.E.M., *P<0.05 (two-tailed unpaired Student's *t*-test)

Bacterial moieties are known to induce the production of ROS in host cells through TLR pathway.^24^ TLRs activate NADPH oxidases and also upregulate mitochondrial ROS production.^26, 27, 28^ We asked whether TLR signaling is responsible for ROS accumulation in the *Tak1*-deficient intestinal epithelium. TLR signaling pathways are mediated through two key adaptor proteins, that is, myeloid differentiation factor 88 (MyD88) and TIR-domain-containing adapter-inducing interferon-*β* (TRIF).^29^ Among them, TLR-MyD88 pathway is implicated in the activation of ROS production.^26, 27^ To test the involvement of TLR-MyD88 signaling in *Tak1* deficiency-induced ROS, we utilized the inducible *Myd88*-deficient system.^30^ We generated mice harboring compound inducible deletion of *Tak1* and *Myd88* on a background of Tnfr1^*−/−*^ (Tak1^IE-IKO^, Myd88^IE-IKO^ Tnfr1^*−/−*^). *Myd88* mRNAs in the small intestine were reduced after tamoxifen injection (Supplementary Figure S3A). We examined ROS levels in Tak1^IE-IKO^, Myd88^IE-IKO^ Tnfr1^*−/−*^ and *Myd88* heterozygous inducible deletion littermate (Tak1^IE-IKO^, Myd88^Het^ Tnfr1^*−/−*^) mice. *Myd88* heterozygous intestinal epithelium exhibited increased ROS similar to *Tak1* single-deficient intestinal epithelium (Figure 4b), but homozygous deletion of *Myd88* alleviated the accumulation of ROS (Figures 4a and b). TUNEL-positive and cleaved caspase-3-positive cells were also decreased but not completely diminished in Tak1^IE-IKO^, Myd88^IE-IKO^ Tnfr1^*−/−*^ mice (Figures 4c and d, and Supplementary Figures S3B and S3C). Thus, *Myd88* deletion resembles the antibiotic treatment, suggesting that commensal bacteria-induced TLR-MyD88 signaling is one of the major pathways to induce excessive ROS accumulation in the *Tak1*-deficient intestinal epithelium. We note here that this partial prevention of ROS accumulation by antibiotic treatment or *Myd88* deletion only marginally improved intestinal injury (Supplementary Figures S2D and S3D), suggesting that additional mechanisms are also involved in tissue injury in the *Tak1*-deficient intestinal epithelium.

### Paneth cell loss is independent of gut bacteria or MyD88

**Figure 5:**
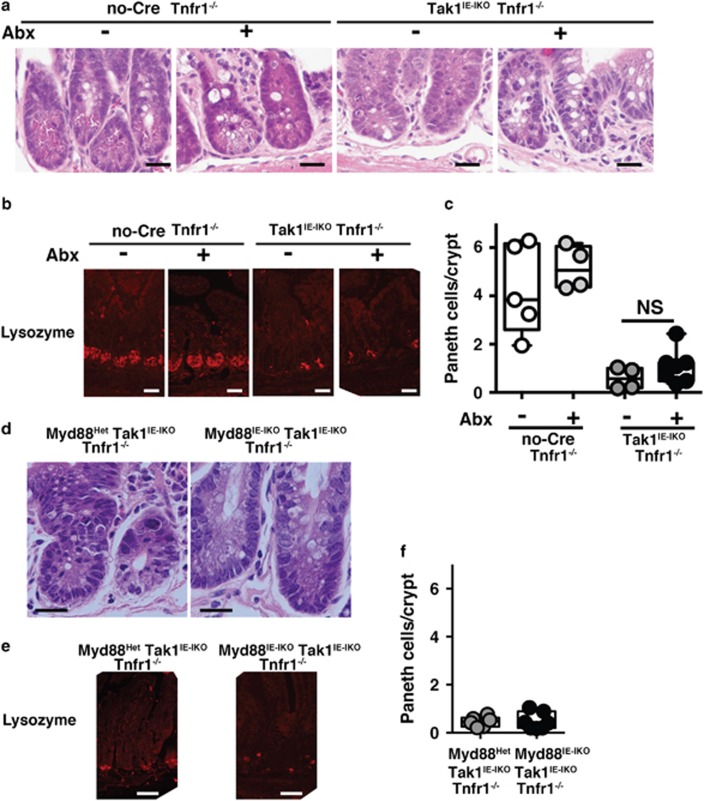
Antibiotic treatment or Myd88 deletion does not block Paneth cell loss. (**a**) H&E staining and (**b**) immunofluorescence staining of lysozyme in the crypts of ileum with and without antibiotic treatment at days 7–10 after tamoxifen injection. Scale bars, (**a**) 20 *μ*m; (**b**) 40 *μ*m. (**c**) Quantification of lysozyme staining-positive cells. More than 100 crypts per mouse were counted. Control Tnfr1^*−/−*^ mice; without (*n*=5) and with (*n*=5) antibiotics. Tak1^IE-IKO^ Tnfr1^*−/−*^ mice; without (*n*=4) and with (*n*=7) antibiotics. All data points are shown in the box and whisker plots: median and distribution of 50% of values are shown in the box: whiskers indicate distribution of minimum and maximum values. NS, not significant (one-way ANOVA). (**d**) H&E staining and (**e**) immunofluorescence staining of lysozyme of *Myd88* heterozygous or *Myd88* homozygous deletion Tak1^IE-IKO^ Tnfr1^*−/−*^ ileum crypts at days 10–12 after tamoxifen injection. Scale bars, (**d**) 20 *μ*m; (**e**) 50 *μ*m. (**f**) Quantification of (**e**) (more than 100 crypts per mouse). *Myd88* heterozygous deletion Tak1^IE-IKO^ Tnfr1^*−/−*^ mice; *n*=6; *Myd88* homozygous deletion Tak1^IE-IKO^ Tnfr1^*−/−*^ mice; *n*=7. All data points are shown in the box and whisker plots: median and distribution of 50% of values are shown in the box: whiskers indicate distribution of minimum and maximum values. NS, not significant (one-way ANOVA)

If highly accumulated ROS are the cause of Paneth cell loss, antibiotic treatment or *Myd88* deletion should block loss of Paneth cells in the *Tak1*-deficient intestinal epithelium. However, hematoxylin and eosin (H&E) staining revealed that granulated cells in the base of crypt were still not observed in the antibiotic-treated Tak1^IE-IKO^ Tnfr1^*−/−*^ intestinal epithelium (Figure 5a). We counted the number of Paneth cells in each crypt by using the Paneth cell marker, lysozyme, which we could clearly detect and visualize individual Paneth cells (see Supplementary Figure S1A). Only a few crypt base cells were detected as positive for lysozyme in Tak1^IE-IKO^ Tnfr1^*−/−*^ (Figures 5b and c). Similarly, Paneth cells were not increased in Tak1^IE-IKO^ Myd88^IE-IKO^ Tnfr1^*−/−*^ intestinal crypts compared with *Myd88* heterozygous deletion mice (Figures 5d–f). We have previously reported that treatment with a ROS scavenger, butylated hydroxyanisole, can diminish ROS-positive cells and reduces cell death in the Tak1^IE-IKO^ Tnfr1^*−/−*^ intestinal epithelium.^3^ However, Paneth cell loss was still observed in the butylated hydroxyanisole-treated Tak1^IE-IKO^ Tnfr1^*−/−*^ intestine (Supplementary Figure S4A). Thus, accumulated ROS are not the cause of Paneth cell loss in the *Tak1*-deficient intestinal epithelium. These results suggest that commensal bacteria are causally involved in increased ROS in the *Tak1*-deficient intestinal epithelium, whereas Paneth cells are depleted through a bacteria-ROS-independent mechanism.

### RIPK3-dependent cell death is involved in Paneth cell loss and is the cause of ROS accumulation

**Figure 6:**
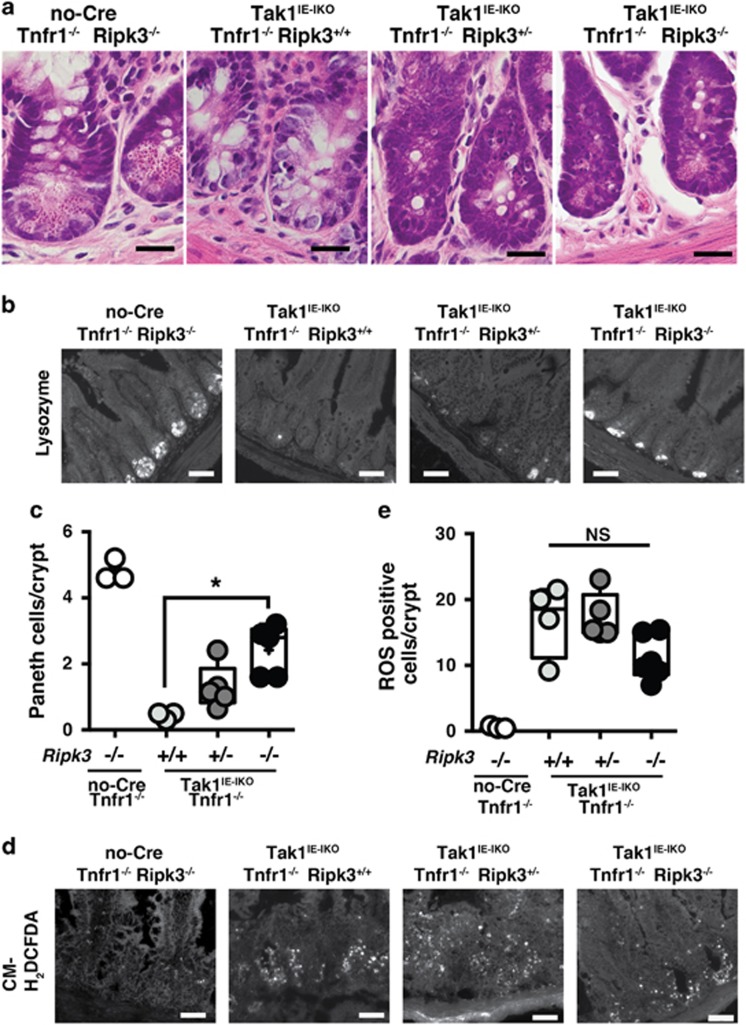
Ripk3 deletion partially rescues Paneth cells loss and marginally reduces ROS accumulation. (**a**) H&E staining and (**b**) immunofluorescence staining of lysozyme of the indicated mouse genotype ileum crypts at day 14 after tamoxifen injection. Scale bars, (**a**) 20 *μ*m; (**b**) 50 *μ*m. (**c**) Quantification of (**b**) (more than 50 crypts per mouse). no-Cre Tnfr1^*−/−*^ Ripk3^*−/−*^, *n*=3; Tak1^IE-IKO^ Tnfr1^*−/−*^ Ripk3^+/+^; *n*=3; Tak1^IE-IKO^ Tnfr1^*−/−*^ Ripk3^+/-^; *n*=5; Tak1^IE-IKO^ Tnfr1^*−/−*^ Ripk3^*−/−*^; *n*=5. All data points are shown in the box and whisker plots: median and distribution of 50% of values are shown in the box: whiskers indicate distribution of minimum and maximum values. *P<0.05 (one-way ANOVA). (**d**) ROS dye staining of the ileum crypts at day 14 after tamoxifen injection. Scale bars, 50 μm. (**e**) Quantification of (**d**) (more than 30 crypts per mouse). no-Cre Tnfr1^*−/−*^ Ripk3^*−/−*^, *n*=3; Tak1^IE-IKO^ Tnfr1^*−/−*^ Ripk3^+/+^; *n*=4; Tak1^IE-IKO^ Tnfr1^*−/−*^ Ripk3^+/-^; *n*=6; Tak1^IE-IKO^ Tnfr1^*−/−*^ Ripk3^*−/−*^; *n*=5. All data points are shown in the box and whisker plots: median and distribution of 50% of values are shown in the box: whiskers indicate distribution of minimum and maximum values. NS, not significant (one-way ANOVA)

Our results above demonstrate that Paneth cell depletion is not due to bacteria-induced ROS. However, Paneth cells were depleted within a period shorter than their normal life span, and *Tak1* deletion structurally disrupts Paneth cells (see Figure 1c). Thus, the cause of Paneth cell loss is still likely due to cell death. Ablation of *Tak1* is known to primarily induce apoptotic cell death;^7^ however, it is also implicated in induction of necroptosis.^8, 9, 10, 11^ Intestinal epithelial-specific deletion of *Tak1* could potentially induce apoptosis and/or necroptosis in Paneth cells. Interestingly, it was reported that intestinal epithelial-specific deletion of necroptosis inhibitors such as caspase 8 and its activator Fas-associated protein with death domain (FADD) induces Paneth cells loss.^31, 32^ This might suggest that Paneth cells are sensitive to necroptosis. Necroptosis is morphologically indistinguishable from necrosis but characterized by a specific feature, dependency on a protein kinase, receptor interacted protein kinase 3 (RIPK3).^33^ RIPK3 is expressed in Paneth cells.^31^ To determine whether Paneth cell loss in the *Tak1*-deficient intestinal epithelium is caused by necroptosis, we generated intestinal epithelial-specific deletion of *Tak1* Tnfr1^*−/−*^ on a background of Ripk3^*−/−*^ mice (Tak1^IE-IKO^ Tnfr1^*−/−*^ Ripk3^*−/−*^). We analyzed and compared the ileum of no-Cre Tnfr1^*−/−*^ Ripk3^*−/−*^ (control), Tak1^IE-IKO^ Tnfr1^*−/−*^ Ripk3^+/+^, Tak1^IE-IKO^ Tnfr1^*−/−*^ Ripk3^+^^/*−*^ and Tak1^IE-IKO^ Tnfr1^*−/−*^ Ripk3^*−/−*^. Intestinal injury was still observed in Tak1^IE-IKO^ Tnfr1^*−/−*^ Ripk3^*−/−*^ mice (Figure 6a). However, we found that Paneth cell loss was partially blocked by *Ripk3* deletion (Figures 6b and c). These suggest that Paneth cells in the *Tak1*-deficient intestinal epithelium were depleted at least partially by necroptosis. Finally, we examined whether this partial restoration of Paneth cells could alleviate ROS. We found that the level of ROS was marginally decreased by deletion of *Ripk3* (Figures 6d and e). Although there is a trend of ROS reduction by *Ripk3* deletion, no statistical significance is observed (Figure 6e). TUNEL-positive cells were not observably altered by *Ripk3* deletion (Supplementary Figures S4B and C). This suggests that most of cell the death observed in the *Tak1*-deficient intestinal epithelium is not dependent on RIPK3 but Paneth cell loss is selectively associated with this form of cell death. Collectively, TAK1 regulates Paneth cell loss and bacteria-induced ROS accumulation through two independent mechanisms. However, the moderate reduction of ROS by deletion of *Ripk3* suggests the possibility that Paneth cell loss is in part causally associated with ROS accumulation.

## Discussion

Paneth cells are unique epithelial cells in the small intestine, which are raised from intestinal epithelial stem cells as are other intestinal epithelial cell types but migrate downward while all other cell types migrate upwards. Paneth cells are specialized to secrete antimicrobial peptides and enzymes to control microbiota in the small intestinal crypts. Paneth cells are also visually unique in histological analysis, in which eosinophilic large granules occupy most of the cytoplasm. Destruction of Paneth cells is often histologically observed in ileitis from patients having one type of IBD, Crohn's disease.^18, 19^ Given their importance in gut microbiota homeostasis, disrupted Paneth cells are likely to be causally associated with ileitis. Indeed, Paneth cell loss has recently been implicated in the initiation of intestinal inflammation.^34^ Thus, determination of the mechanism of how Paneth cells are maintained is important for better understanding of IBD pathology and treatment. Paneth cell loss has been reported in several genetically engineered mouse models. Most intriguingly, intestinal epithelium-specific deletion of *caspase 8* or its activator, *Fadd*, which are inhibitors of necroptosis, depletes Paneth cells.^31, 32^ This loss of Paneth cells is rescued by deletion of necroptosis mediator, *Ripk3*. Furthermore, RIPK3 is increased in the intestine of IBD patient samples.^31^ Thus, activation of necroptosis is likely to be one of the causes of pathological Paneth cell loss. However, the pathway of how necroptosis is activated in the intestinal epithelium is not clear. Our current study reveals that TAK1 is required for the prevention of Paneth cell death. As this cell death is partially prevented by deletion of a necroptosis mediator *Ripk3*, *Tak1* deletion causes Paneth loss in part through induction of necroptosis. TAK1 is a protein kinase mediating inflammatory intracellular signaling pathways leading to NF-*κ*B and AP-1, which is activated by a variety of inflammatory stimuli including TNF, IL-1 and Toll-like receptor ligands. In these signaling pathways, another protein kinase RIPK1 is ubiquitilyated, which serves as a scaffold of signaling molecules including TAK1.^35^ Both RIPK1 and TAK1 are essential molecules in these inflammatory signal transduction pathways. Recently, intestinal epithelial-specific deletion of *Ripk1* was reported to deplete Paneth cells.^36, 37^ Thus, deletion of either *Tak1* or *Ripk1* results in Paneth cell loss. This raises the possibility that impairment of inflammatory signaling causes Paneth cell loss. Given that intestinal epithelium is constantly exposed to gut bacteria and immune cell-derived cytokines, it may not be surprising that proper inflammatory signaling from bacteria and cytokines is involved in the maintenance of Paneth cells. Homeostatic intestinal inflammatory signaling may be one of the key factors to maintain Paneth cells through preventing RIPK3-dependent necroptosis.

In the *Tak1*-deficient intestinal epithelium, ROS are highly accumulated and the intestinal epithelium is severely damaged. Our results demonstrate that gut bacteria cause ROS accumulation in the *Tak1*-deficient intestinal epithelium. Because inhibition of Paneth cell loss slightly reduces ROS accumulation in the *Tak1*-deficient intestinal epithelium, Paneth cell loss may disrupt normal commensal microbiota, which may be involved in ROS accumulation. However, ROS accumulation and cell death in the *Tak1*-deficient intestinal epithelium seem to be much more pronounced compared with other genetically engineered mouse models harboring Paneth cell depletion. For example, *caspase 8* or *Fadd* deletion gradually induces ileitis in a non-inducible version of intestinal epithelium-specific gene deletion system,^31, 32^ whereas the same deletion system causes severe tissue damage and neonatal lethality when *Tak1* is deleted.^13^ This suggests that additional mechanisms are involved in the ROS-induced tissue injury by *Tak1* deletion. *Tak1* deletion has been shown to reduce the capacity of cellular antioxidant systems through downregulation of antioxidant transcription factors such as NF-*κ*B, AP-1 and Nrf2.^3, 5^ We previously showed that *Tak1* deletion downregulates the levels of Nrf2 and its target antioxidant enzyme, (NAD(P)H dehydrogenase 1 (NQO1).^3^ Thus, the impaired antioxidant system may contribute to the high accumulation of ROS in the *Tak1*-deficient intestinal epithelium. Our results collectively demonstrate that basal activity of TAK1 in the normal intestine is critical in intestinal homeostasis by preventing Paneth cell loss and unattended accumulation of bacteria-induced ROS.

## Materials and Methods

### Mice

Mice carrying a floxed Map3k7 allele (*Tak1*^*fl/fl*^)^38^ were backcrossed to C57BL/6 mice for at least seven generations. *Tnfr1*-deficient (*Tnfr1*^*−/−*^),^39^ Myd88-floxed (*Myd88*^*fl/fl*^)^30^ and an intestinal epithelium-specific deleter (*villin.Cre*)^40^ mice with a C57BL/6 background were from The Jackson Laboratory. The inducible intestinal epithelium-specific deleter (*villin.CreER*^*T2*^) (a gift from Dr. Robine)^41^ and *Ripk3*-deficient (*Ripk3*^*−/−*^) (a gift from Dr. Dixit)^42^ were also used. We generated constitutive and inducible versions of intestinal epithelium-specific *Tak1* (*villin.Cre Tak1*^*fl/fl*^, TAK1^IE-KO^; and *villin.CreER*^*T2*^ *Tak1*^*fl/fl*^, TAK1^IE-IKO^, respectively) on a *Tnfr1*^*−/−*^ or *Tnfr1*^*−/−*^*Ripk3*^*−/−*^ background. Intestinal epithelium-specific compound deletion of *Tak1* and *Myd88* (*villin.CreER*^*T2*^ *Tak1*^*fl/fl*^*Myd88*^*fl/fl*^, TAK1 Myd88^IE-IKO^) mice were generated on a *Tnfr1*^*−/−*^ background. Littermate control mice (no-Cre *Tak1*^*fl/fl*^ or *villin.CreER*^T2^ *Tak1*^*fl/+*^ on a *Tnfr1*^*−/−*^ or *Tnfr1*^*−/−*^*Ripk3*^*−/−*^ background) were included in all experiments, but some other litter control mice were also used. All control (*Tak1* wild-type or heterozygous deletion) mice exhibited no ROS accumulation and four to six Paneth cells were observed in each crypt. To induce gene deletion, 6–12- week-old mice were given intraperitoneal injections of tamoxifen (1 mg per mouse, approximately 20 g body weight, per day) for three to five consecutive days. The first day of tamoxifen injection is herein referred to as day 1. For antibiotic treatment, the antibiotic cocktail consisting of ampicillin (1 g/l), vancomycin (0.5 g/l), neomycin sulfate (1 g/l) and metronidazole (1 g/l)^25^ was added to the drinking water of 6–8 week-old mice for 4 weeks prior to the tamoxifen injected. The antibiotic treatment was continued during and after the tamoxifen injection until the end of experiments. Mice were maintained in ventilated cages at the specific pathogen-free animal facility and fed regular chow diet. All animal experiments were conducted with the approval of the North Carolina State University Institutional Animal Care and Use Committee. All efforts were made to minimize animal suffering.

### Histology and immunofluorescence staining

For H&E staining, a part of ileum was fixed in 4% paraformaldehyde and embedded into paraffin, and cross sections were stained by H&E. Sections are scored in a blinded manner on the scale from 0 to 4, based on the degree of lamina propria mononuclear cell infiltration, crypt hyperplasia, goblet cell depletion and architectural distortion described previously.^13, 43^ To detect intestinal ROS, ileums were embedded optimum cutting temperature compound and frozen immediately. Cryosections (8 *μ*m) were incubated with the ROS staining dye (CM-H_2_DCFDA, Life Technologies, Waltham, MA, USA) for 30 min at room temperature. To detect cell death, paraffin-embedded sections were used for DeadEnd Fluorometric TUNEL staining (Promega, Madison, WI, USA). To detect Paneth cells, immunofluorescence staining of lysozyme and Alcian blue staining were performed. For immunofluorescence staining of lysozyme, 4% paraformaldehyde-fixed paraffin sections were rehydrated, heat-induced antigen retrieval was performed in a citrate buffer (10 mM citric acid, 0.05% Tween 20, pH 6.0), and the sections were stained using the muramidase (lysozyme) primary antibody (1:200, Novocastra, Leica, Buffalo Grove, IL, USA) overnight at 4 °C. Bound antibodies were visualized by the Alexa Fluor 594 fluorescence dye-conjugated secondary antibody. Paraffin-embedded sections fixed with 4% paraformaldehyde were used for Alcian blue staining. Some sections were counterstained with Schiffs reagent. To determine cell proliferation, thymidine analog, 5-ethynyl-2'-deoxyuridine (EdU) (1 mg per mouse) was injected 4 h prior to euthanasia, cryosections fixed with 4% paraformaldehyde were prepared. EdU incorporation was visualized by Click-iT EdU Alexa Fluor 488 Imaging Kit (Life Technologies). For immunofluorescence staining of cleaved caspase 3, cryosections fixed with 4% paraformaldehyde were incubated with primary antibodies against cleaved caspase 3 (Asp175, 1:200, Cell Signaling, Danvers, MA, USA). Bound antibodies were visualized by the Alexa Fluor 488 fluorescence dye-conjugated secondary antibody (1:1000, Life Technologies). Nuclei were counterstained with DAPI. Images were visualized using a fluorescent microscope (BX41; Olympus, Waltham, MA, USA) controlled by the CellSens imaging software (Olympus). Random portions of the intestine were selected and images were visualized and photographed using the same exposure times. To quantify the positive stained cells, we pick five to six areas from more than three different cross sections per animal, and counted cells in each crypt. Any non-specific stainings that did not have nuclear DAPI staining were removed.

### Immunoblotting analysis of intestinal epithelial cells

The small intestine was harvested and flushed with phosphate buffer saline. One end of the intestine was tied off, filled with Hanks' Balanced Salt Solution (HBSS, Sigma, St. Louis, MO, USA) containing 10 mM EDTA and incubated in a phosphate buffer saline bath at 37 °C for 5–10 min. The contents (intestinal epithelial cells) were collected and lysed in a cell extraction buffer containing 20 mM HEPES (pH 7.4), 150 mM NaCl, 12.5 mM *β*-glycerophosphate, 1.5 mM MgCl2, 2 mM EGTA, 10 mM NaF, 2 mM DTT, 1 mM Na3VO4, 1 mM phenylmethylsulfonyl fluoride, 20 *μ*M aprotinin and 0.5% Triton X-100. Proteins were electrophoresed on SDS-PAGE and transferred to Hypond-P membranes (GE Healthcare, Pittsburgh, PA, USA). The membranes were immunoblotted with anti-TAK1^44^ and *β*-actin (AC-15, Sigma), and the bound antibodies were visualized with horseradish peroxidase-conjugated antibodies against rabbit or mouse IgG using the ECL Western blotting system (GE Healthcare).

### mRNA and bacteria DNA detection by real-time PCR

RNA was isolated by using RNeasy Kit (Qiagen), and *Myd88* mRNA was determined by real-time PCR. Feces bacterial DNA was isolated using QIAamp DNA Stool Mini Kit (Qiagen, Valencia, CA, USA) and purified DNAs were analyzed by using two different bacterial ribosome S16 universal primers^45, 46^ and a primer set for *Bacteroidetes*.^46^ Primers used were *Myd88*, 5′-CACCTGTGTCTGGTCCATT-3′ 5′-AGGCTGAGTGCAAACTTG-3′:^47^ ribosome S16 universal-1, 5′-ACTCCTACGGGAGGCAGCAG-3′; 5′-ATTACCGCGGCTGCTGG-3′: ribosome S16 universal-2, 5′-GTGSTGCAYGGYTGTCGTCA-3′ 5′-ACGTCRTCCMCACCTTCCTC-3′: *Bacteroidetes*, 5′-GGARCATGTGGTTTAATTCGATGAT-3′; 5′-AGCTGACGACAACCATGCAG-3′. mRNA levels were normalized to *Gapdh* mRNA. Primers for *Gapdh*, 5′-GAAGGTCGCTGTGAACGGA-3′; 5′-GTTAGTGGGGTCTCGCTCCT-3′. Bacterial DNA levels were normalized to feces weight.

### Statistical analysis

All experiments were conducted using at least three mice and the results are confirmed by at least three separately performed experiments. The box plots show medians (line), lower and upper quartiles (boxes), 10th and 90th percentiles (whiskers) and outliers. The column graphs represent the mean±the standard deviation. Differences between experimental groups were assessed for significance by using the one-way ANOVA with Tukey's multiple comparisons test or the unpaired Students *t*-test (two-tailed) with equal distributions.

## Abbreviations

FADD: Fas-associated protein with death domain
IBD: inflammatory bowel disease
MAP3K: mitogen-activated protein kinase kinase kinase
Myd88: myeloid differentiation factor 88
NQO1: NADPH quinone oxidoreductase-1
Nrf2: Nuclear factor erythroid 2 (NF-E2)-related factor 2
P: postnatal day
RIPK3: receptor-interacting protein kinase 3
ROS: reactive oxygen species
TLR: Toll-like receptor
TNFR1: tumor necrosis factor 1 receptor 1
TRIF: TIR-domain-containing adapter-inducing interferon-*β*
TUNEL: terminal deoxynucleotidyl transferase dUTP terminal nick-end labeling

## References

[bib1] Mihaly SR, Ninomiya-Tsuji J, Morioka S. TAK1 control of cell death. Cell Death Differ 2014; 21: 1667–1676. 25146924 10.1038/cdd.2014.123 PMC4211365

[bib2] Sen CK, Packer L. Antioxidant and redox regulation of gene transcription. FASEB J 1996; 10: 709–720. 8635688 10.1096/fasebj.10.7.8635688

[bib3] Kajino-Sakamoto R, Omori E, Nighot PK, Blikslager AT, Matsumoto K, Ninomiya-Tsuji J. TGF-beta-activated kinase 1 signaling maintains intestinal integrity by preventing accumulation of reactive oxygen species in the intestinal epithelium. J Immunol 2010; 185: 4729–4737. 20855879 10.4049/jimmunol.0903587 PMC3064262

[bib4] Morioka S, Omori E, Kajino T, Kajino-Sakamoto R, Matsumoto K, Ninomiya-Tsuji J. TAK1 kinase determines TRAIL sensitivity by modulating reactive oxygen species and cIAP. Oncogene 2009; 28: 2257–2265. 19421137 10.1038/onc.2009.110 PMC2796077

[bib5] Omori E, Morioka S, Matsumoto K, Ninomiya-Tsuji J. TAK1 regulates reactive oxygen species and cell death in keratinocytes, which is essential for skin integrity. J Biol Chem 2008; 283: 26161–26168. 18606807 10.1074/jbc.M804513200 PMC2533783

[bib6] Omori E, Matsumoto K, Zhu S, Smart RC, Ninomiya-Tsuji J. Ablation of TAK1 upregulates reactive oxygen species and selectively kills tumor cells. Cancer Res 2010; 70: 8417–8425. 20959492 10.1158/0008-5472.CAN-10-1227 PMC2970664

[bib7] Morioka S, Broglie P, Omori E, Ikeda Y, Takaesu G, Matsumoto K et al. TAK1 kinase switches cell fate from apoptosis to necrosis following TNF stimulation. J Cell Biol 2014; 204: 607–623. 24535827 10.1083/jcb.201305070 PMC3926964

[bib8] Lamothe B, Lai Y, Xie M, Schneider MD, Darnay BG. TAK1 is essential for osteoclast differentiation and is an important modulator of cell death by apoptosis and necroptosis. Mol Cell Biol 2013; 33: 582–595. 23166301 10.1128/MCB.01225-12 PMC3554219

[bib9] Arslan SC, Scheidereit C. The prevalence of TNFalpha-induced necrosis over apoptosis is determined by TAK1-RIP1 interplay. PLoS ONE 2011; 6: e26069. 22016814 10.1371/journal.pone.0026069 PMC3189922

[bib10] Vanlangenakker N, Vanden Berghe T, Bogaert P, Laukens B, Zobel K, Deshayes K et al. cIAP1 and TAK1 protect cells from TNF-induced necrosis by preventing RIP1/RIP3-dependent reactive oxygen species production. Cell Death Differ 2011; 18: 656–665. 21052097 10.1038/cdd.2010.138 PMC3131911

[bib11] Vucur M, Reisinger F, Gautheron J, Janssen J, Roderburg C, Cardenas DV et al. RIP3 inhibits inflammatory hepatocarcinogenesis but promotes cholestasis by controlling caspase-8- and JNK-dependent compensatory cell proliferation. Cell Rep 2013; 4: 776–790. 23972991 10.1016/j.celrep.2013.07.035

[bib12] Omori E, Matsumoto K, Ninomiya-Tsuji J. Non-canonical β-catenin degradation mediates reactive oxygen species-induced epidermal cell death. Oncogene 2011; 30: 3336–3344. 21383695 10.1038/onc.2011.49 PMC3131442

[bib13] Kajino-Sakamoto R, Inagaki M, Lippert E, Akira S, Robine S, Matsumoto K et al. Enterocyte-derived TAK1 signaling prevents epithelium apoptosis and the development of ileitis and colitis. J Immunol 2008; 181: 1143–1152. 18606667 10.4049/jimmunol.181.2.1143 PMC3065656

[bib14] Kim JY, Kajino-Sakamoto R, Omori E, Jobin C, Ninomiya-Tsuji J. Intestinal epithelial-derived TAK1 signaling is essential for cytoprotection against chemical-induced colitis. PLoS ONE 2009; 4: e4561. 19234607 10.1371/journal.pone.0004561 PMC2642721

[bib15] Bevins CL, Salzman NH. Paneth cells, antimicrobial peptides and maintenance of intestinal homeostasis. Nat Rev Microbiol 2011; 9: 356–368. 21423246 10.1038/nrmicro2546

[bib16] Sancho E, Batlle E, Clevers H. Live and let die in the intestinal epithelium. Curr Opin Cell Biol 2003; 15: 763–770. 14644203 10.1016/j.ceb.2003.10.012

[bib17] Ireland H, Houghton C, Howard L, Winton DJ. Cellular inheritance of a Cre-activated reporter gene to determine Paneth cell longevity in the murine small intestine. Dev Dyn 2005; 233: 1332–1336. 15937933 10.1002/dvdy.20446

[bib18] Xavier RJ, Podolsky DK. Unravelling the pathogenesis of inflammatory bowel disease. Nature 2007; 448: 427–434. 17653185 10.1038/nature06005

[bib19] Kaser A, Zeissig S, Blumberg RS. Inflammatory bowel disease. Annu Rev Immunol 2010; 28: 573–621. 20192811 10.1146/annurev-immunol-030409-101225 PMC4620040

[bib20] Bry L, Falk P, Huttner K, Ouellette A, Midtvedt T, Gordon JI. Paneth cell differentiation in the developing intestine of normal and transgenic mice. Proc Natl Acad Sci USA 1994; 91: 10335–10339. 7937951 10.1073/pnas.91.22.10335 PMC45014

[bib21] McGuckin MA, Linden SK, Sutton P, Florin TH. Mucin dynamics and enteric pathogens. Nat Rev Microbiol 2011; 9: 265–278. 21407243 10.1038/nrmicro2538

[bib22] Halliwell B, Whiteman M. Measuring reactive species and oxidative damage *in vivo* and in cell culture: how should you do it and what do the results mean? Br J Pharmacol 2004; 142: 231–255. 15155533 10.1038/sj.bjp.0705776 PMC1574951

[bib23] Liu Y, Collins C, Kiosses WB, Murray AM, Joshi M, Shepherd TR et al. A novel pathway spatiotemporally activates Rac1 and redox signaling in response to fluid shear stress. J Cell Biol 2013; 201: 863–873. 23733346 10.1083/jcb.201207115 PMC3678169

[bib24] Lambeth JD, Neish AS. Nox enzymes and new thinking on reactive oxygen: a double-edged sword revisited. Annu Rev Pathol 2014; 9: 119–145. 24050626 10.1146/annurev-pathol-012513-104651

[bib25] Rakoff-Nahoum S, Paglino J, Eslami-Varzaneh F, Edberg S, Medzhitov R. Recognition of commensal microflora by toll-like receptors is required for intestinal homeostasis. Cell 2004; 118: 229–241. 15260992 10.1016/j.cell.2004.07.002

[bib26] Lipinski S, Till A, Sina C, Arlt A, Grasberger H, Schreiber S et al. DUOX2-derived reactive oxygen species are effectors of NOD2-mediated antibacterial responses. J Cell Sci 2009; 122: 3522–3530. 19759286 10.1242/jcs.050690

[bib27] Laroux FS, Romero X, Wetzler L, Engel P, Terhorst C. Cutting edge: MyD88 controls phagocyte NADPH oxidase function and killing of gram-negative bacteria. J Immunol 2005; 175: 5596–5600. 16237045 10.4049/jimmunol.175.9.5596

[bib28] West AP, Brodsky IE, Rahner C, Woo DK, Erdjument-Bromage H, Tempst P et al. TLR signalling augments macrophage bactericidal activity through mitochondrial ROS. Nature 2011; 472: 476–480. 21525932 10.1038/nature09973 PMC3460538

[bib29] Kawai T, Akira S. The role of pattern-recognition receptors in innate immunity: update on Toll-like receptors. Nat Immunol 2010; 11: 373–384. 20404851 10.1038/ni.1863

[bib30] Hou B, Reizis B, DeFranco AL. Toll-like receptors activate innate and adaptive immunity by using dendritic cell-intrinsic and -extrinsic mechanisms. Immunity 2008; 29: 272–282. 18656388 10.1016/j.immuni.2008.05.016 PMC2847796

[bib31] Gunther C, Martini E, Wittkopf N, Amann K, Weigmann B, Neumann H et al. Caspase-8 regulates TNF-alpha-induced epithelial necroptosis and terminal ileitis. Nature 2011; 477: 335–339. 21921917 10.1038/nature10400 PMC3373730

[bib32] Welz PS, Wullaert A, Vlantis K, Kondylis V, Fernandez-Majada V, Ermolaeva M et al. FADD prevents RIP3-mediated epithelial cell necrosis and chronic intestinal inflammation. Nature 2011; 477: 330–334. 21804564 10.1038/nature10273

[bib33] Vandenabeele P, Galluzzi L, Vanden Berghe T, Kroemer G. Molecular mechanisms of necroptosis: an ordered cellular explosion. Nat Rev Mol Cell Biol 2010; 11: 700–714. 20823910 10.1038/nrm2970

[bib34] Adolph TE, Tomczak MF, Niederreiter L, Ko HJ, Bock J, Martinez-Naves E et al. Paneth cells as a site of origin for intestinal inflammation. Nature 2013; 503: 272–276. 24089213 10.1038/nature12599 PMC3862182

[bib35] Ea CK, Deng L, Xia ZP, Pineda G, Chen ZJ. Activation of IKK by TNFalpha requires site-specific ubiquitination of RIP1 and polyubiquitin binding by NEMO. Mol Cell 2006; 22: 245–257. 16603398 10.1016/j.molcel.2006.03.026

[bib36] Takahashi N, Vereecke L, Bertrand MJ, Duprez L, Berger SB, Divert T et al. RIPK1 ensures intestinal homeostasis by protecting the epithelium against apoptosis. Nature 2014; 513: 95–99. 25186904 10.1038/nature13706

[bib37] Dannappel M, Vlantis K, Kumari S, Polykratis A, Kim C, Wachsmuth L et al. RIPK1 maintains epithelial homeostasis by inhibiting apoptosis and necroptosis. Nature 2014; 513: 90–94. 25132550 10.1038/nature13608 PMC4206266

[bib38] Sato S, Sanjo H, Takeda K, Ninomiya-Tsuji J, Yamamoto M, Kawai T et al. Essential function for the kinase TAK1 in innate and adaptive immune responses. Nat Immunol 2005; 6: 1087–1095. 16186825 10.1038/ni1255

[bib39] Pfeffer K, Matsuyama T, Kundig TM, Wakeham A, Kishihara K, Shahinian A et al. Mice deficient for the 55 kd tumor necrosis factor receptor are resistant to endotoxic shock, yet succumb to L. monocytogenes infection. Cell 1993; 73: 457–467. 8387893 10.1016/0092-8674(93)90134-c

[bib40] Madison BB, Dunbar L, Qiao XT, Braunstein K, Braunstein E, Gumucio DL. Cis elements of the villin gene control expression in restricted domains of the vertical (crypt) and horizontal (duodenum, cecum) axes of the intestine. J Biol Chem 2002; 277: 33275–33283. 12065599 10.1074/jbc.M204935200

[bib41] el Marjou F, Janssen KP, Chang BH, Li M, Hindie V, Chan L et al. Tissue-specific and inducible Cre-mediated recombination in the gut epithelium. Genesis 2004; 39: 186–193. 15282745 10.1002/gene.20042

[bib42] Newton K, Sun X, Dixit VM. Kinase RIP3 is dispensable for normal NF-κBs, signaling by the B-cell and T-cell receptors, tumor necrosis factor receptor 1, and Toll-like receptors 2 and 4. Mol Cell Biol 2004; 24: 1464–1469. 14749364 10.1128/MCB.24.4.1464-1469.2004 PMC344190

[bib43] Rath HC, Herfarth HH, Ikeda JS, Grenther WB, Hamm TE Jr., Balish E et al. Normal luminal bacteria, especially Bacteroides species, mediate chronic colitis, gastritis, and arthritis in HLA-B27/human beta2 microglobulin transgenic rats. J Clin Invest 1996; 98: 945–953. 8770866 10.1172/JCI118878 PMC507509

[bib44] Ninomiya-Tsuji J, Kishimoto K, Hiyama A, Inoue J, Cao Z, Matsumoto K. The kinase TAK1 can activate the NIK-IκB as well as the MAP kinase cascade in the IL-1 signalling pathway. Nature 1999; 398: 252–256. 10094049 10.1038/18465

[bib45] Maeda H, Fujimoto C, Haruki Y, Maeda T, Kokeguchi S, Petelin M et al. Quantitative real-time PCR using TaqMan and SYBR Green for Actinobacillus actinomycetemcomitans, Porphyromonas gingivalis, Prevotella intermedia, tetQ gene and total bacteria. FEMS Immunol Med Microbiol 2003; 39: 81–86. 14557000 10.1016/S0928-8244(03)00224-4

[bib46] Guo X, Xia X, Tang R, Zhou J, Zhao H, Wang K. Development of a real-time PCR method for Firmicutes and Bacteroidetes in faeces and its application to quantify intestinal population of obese and lean pigs. Lett Appl Microbiol 2008; 47: 367–373. 19146523 10.1111/j.1472-765X.2008.02408.x

[bib47] Subramanian M, Thorp E, Hansson GK, Tabas I. Treg-mediated suppression of atherosclerosis requires MYD88 signaling in DCs. J Clin Invest 2013; 123: 179–188. 23257360 10.1172/JCI64617 PMC3533292

