# Supplementary material for: TAK1 regulates Paneth cell integrity partly through blocking necroptosis
Source: Cell Death Dis. 2016 Apr 14;7(4):e2196–. doi: 10.1038/cddis.2016.98 (PMC4855677; doi:10.1038/cddis.2016.98)
Supplement: Supplementary Figures [file cddis201698x1.doc]

**TAK1 regulates Paneth cell integrity partly through blocking necroptosis**

Alicia N. Simmons, Rie Kajino-Sakamoto, and Jun Ninomiya-Tsuji

**Supplementary Figure Legends**

**Figure S1**

**Paneth cells in *Tak1*-deficient intestinal epithelium.**

(A) Enlarged images of lysozyme staining shown in Fig. 1A bottom panels (upper panels). We also show typical lysozyme staining in the adult ileum (bottom panel). At P17, only one or two lysozyme positive cells with not clear granule staining were observed in the ileum in both control *Tnfr1^-/-^* and Tak1^IE-KO^ mice. Scale bars, 10 μm (upper panels), 50 μm (lower panel).

(B) Alcian blue staining of the P17 ileum of no-Cre *Tnfr1^-/-^* and Tak1^IE-KO^ *Tnfr1^-/-^* mice. Arrows indicate cells having weak staining of Alcian blue at the base of crypt. Scale bars, 20 μm.

(C) Intestinal epithelial cells were collected at day 12 after tamoxifen injection and TAK1 protein was analyzed by immunoblotting. β-actin is shown as a loading control. Asterisk indicates a non-specific band. In this *Tak1*-floxed system,[^1^](#_ENREF_1) a truncated version of TAK1 (TAK1Δ) lacking the ATP binding site was produced by the gene deletion but it was expressed at a lower level compared to intake TAK1 presumably due to instability of a mutant protein.

(D) Heterozygous deletion of *Tak1* and inducible Cre expression does not cause Paneth cell loss. H&E staining of the ileum crypts at day 8 after tamoxifen injection. Scale bars, 20 μm.

(E) H&E staining of the colonic crypts at day 4, day 7 and 2 months after the initiation of *Tak1* gene deletion. Arrows indicate cells morphologically showing apoptotic features. Scale bars, 20 μm.

**Figure S2**

**Antibiotic treatment partially rescued cell death and tissue injury in *Tak1*-deficient intestinal epithelium.**

(A) Feces bacteria were reduced by antibiotics. Gut bacteria were assessed in feces from mice (wild type) treated with the antibiotic cocktail or standard water for 5 weeks by using two different universal bacteria 16S ribosomal DNA primers; and a primer set for *Bacteroidetes*.[^3^](#_ENREF_3) Water control, n = 6; antibiotic cocktail treated, n = 3. Means ± SEM. **, p < 0.01.

(B,C) Caspase activation was diminished by bacterial depletion.

(B) Immunofluorescent staining of cleaved caspase 3 at day 14 after tamoxifen injection. Scale bars, 50 µm.

(C) Cells with cleaved caspase 3-positive staining were counted (more than 100 crypts per mouse). 3 mice per treatment group were analyzed. Means ±SEM, **, p < 0.01 (two-tailed unpaired Student’s t test).

(D) Intestinal injury was slightly improved by antibiotic treatment. Histology scores on the scale from 0 to 4 as described in Materials and Methods at day 7-12 after tamoxifen injection are shown. No-Cre Tnfr1^-/-^ without (n = 5) and with (n = 6) antibiotic treatment (Abx); Tak1^IE-IKO^ Tnfr1^-/-^ without (n = 7) and with (n = 7) antibiotic treatment. Means ±SEM. *, p < 0.05 (two-tailed unpaired Student’s t test).

**Figure S3**

***Myd88* deletion partially rescued cell death and tissue injury in *Tak1*-deficient intestinal epithelium.**

(A) Total RNA was isolated from the small intestine at day 10-12 after tamoxifen injection. mRNA levels of Myd88 was assessed by quantitative real-time PCR. *Myd88* heterozygous deletion Tak1^IE-IKO^ Tnfr1^-/-^ mice; n = 4; *Myd88* homozygous deletion Tak1^IE-IKO^ Tnfr1^-/-^ mice; n = 3. Means ±SEM, *p* value of two-tailed unpaired Student’s t test is shown. Myd88 is highly expressed in lamina propria immune cells, which are included in RNA samples but not affected by *villin.CreERT2.* Thus, the reduction of *Myd88* mRNA levels may not be profound.

(B) Immunofluorescent staining of cleaved caspase 3 at day 10-12 after tamoxifen injection. Scale bars, 50 µm.

(C) Cells with cleaved caspase 3-positive staining were counted (more than 100 crypts per mouse). *Myd88* heterozygous deletion Tak1^IE-IKO^ Tnfr1^-/-^ mice; n = 3; *Myd88* homozygous deletion Tak1^IE-IKO^ Tnfr1^-/-^ mice; n = 5. Means ±SEM, **, p < 0.01 (two-tailed unpaired Student’s t test).

(D) Intestinal injury is slightly improved by *Myd88* deletion in *Tak1*-deficient intestinal epithelium. Histology scores on the scale from 0 to 4 as described in Materials and Methods at day 7-12 after tamoxifen injection are shown. Tak1^IE-IKO^ Myd88^Het^ Tnfr1^-/-^ and Tak1^IE-IKO^ Myd88^IE-IKO^ Tnfr1^-/-^, n = 5. Means ±SEM. *, p < 0.05 (two-tailed unpaired Student’s t test).

**Figure S4**

**ROS scavenger did not block Paneth cell loss, and *Ripk3* deletion did not reduce overall cell death in the *Tak1*-deficient intestinal epithelium.**

(A) no-Cre Tnfr1^-/-^ and Tak1^IE-IKO^ Tnfr1^-/-^ mice were fed with normal food or food containing 0.7% butylated hydroxyanisole (BHA) from 1 week prior to the tamoxifen injection, and kept under BHA diet for the entire period of the experiment. Small intestine was analyzed at day 7 after tamoxifen injection. Scale bars, 20 μm.

(B) TUNEL staining of indicated mouse genotype ileum crypts at day 14 after tamoxifen injection. We observed TUNEL stained sections of no-Cre Tnfr1^-/-^ Ripk3^-/-^, n = 3; Tak1^IE-IKO^ Tnfr1^-/-^ Ripk3^+/+^; n = 3 (not shown); Tak1^IE-IKO^ Tnfr1^-/-^ Ripk3^+/-^; n = 6; Tak1^IE-IKO^ Tnfr1^-/-^ Ripk3^-/-^; n = 8. Representative pictures are shown. Scale bars, 50μm.

(C) TUNEL positive cells were quantified. no-Cre Tnfr1^-/-^ Ripk3^-/-^, n = 3; ; Tak1^IE-IKO^ Tnfr1^-/-^ Ripk3^+/-^; n = 6; Tak1^IE-IKO^ Tnfr1^-/-^ Ripk3^-/-^; n = 8. All data points are shown in the box and whisker plots: median and distribution of 50% of values are shown in the box: whiskers indicate distribution of minimum and maximum values. NS, not significant (one-way ANOVA).

1. Sato S, Sanjo H, Takeda K, Ninomiya-Tsuji J, Yamamoto M, Kawai T*, et al.* Essential function for the kinase TAK1 in innate and adaptive immune responses. Nat. Immunol. 2005; **6**: 1087-1095.

2. Maeda H, Fujimoto C, Haruki Y, Maeda T, Kokeguchi S, Petelin M*, et al.* Quantitative real-time PCR using TaqMan and SYBR Green for Actinobacillus actinomycetemcomitans, Porphyromonas gingivalis, Prevotella intermedia, tetQ gene and total bacteria. FEMS Immunol. Med. Microbiol. 2003; **39**: 81-86.

3. Guo X, Xia X, Tang R, Zhou J, Zhao H, Wang K. Development of a real-time PCR method for Firmicutes and Bacteroidetes in faeces and its application to quantify intestinal population of obese and lean pigs. Lett. Appl. Microbiol. 2008; **47**: 367-373.
