# Supplementary figures and images for: TAK1 regulates Paneth cell integrity partly through blocking necroptosis
Source: Cell Death Dis. 2016 Apr 14;7(4):e2196–. doi: 10.1038/cddis.2016.98 (PMC4855677; doi:10.1038/cddis.2016.98)

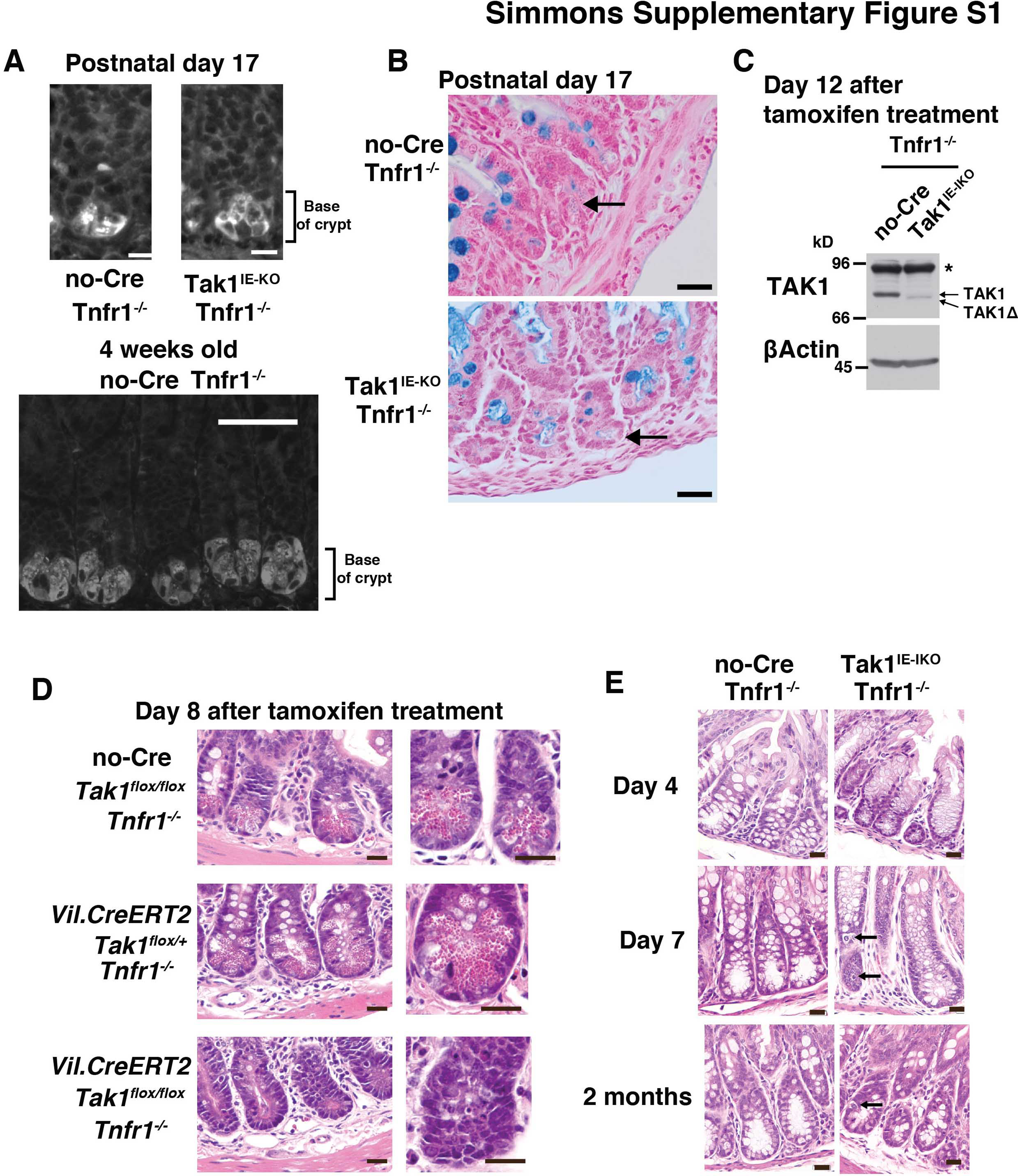

Supplement: Supplementary Figure 1 [file cddis201698x2.tif]

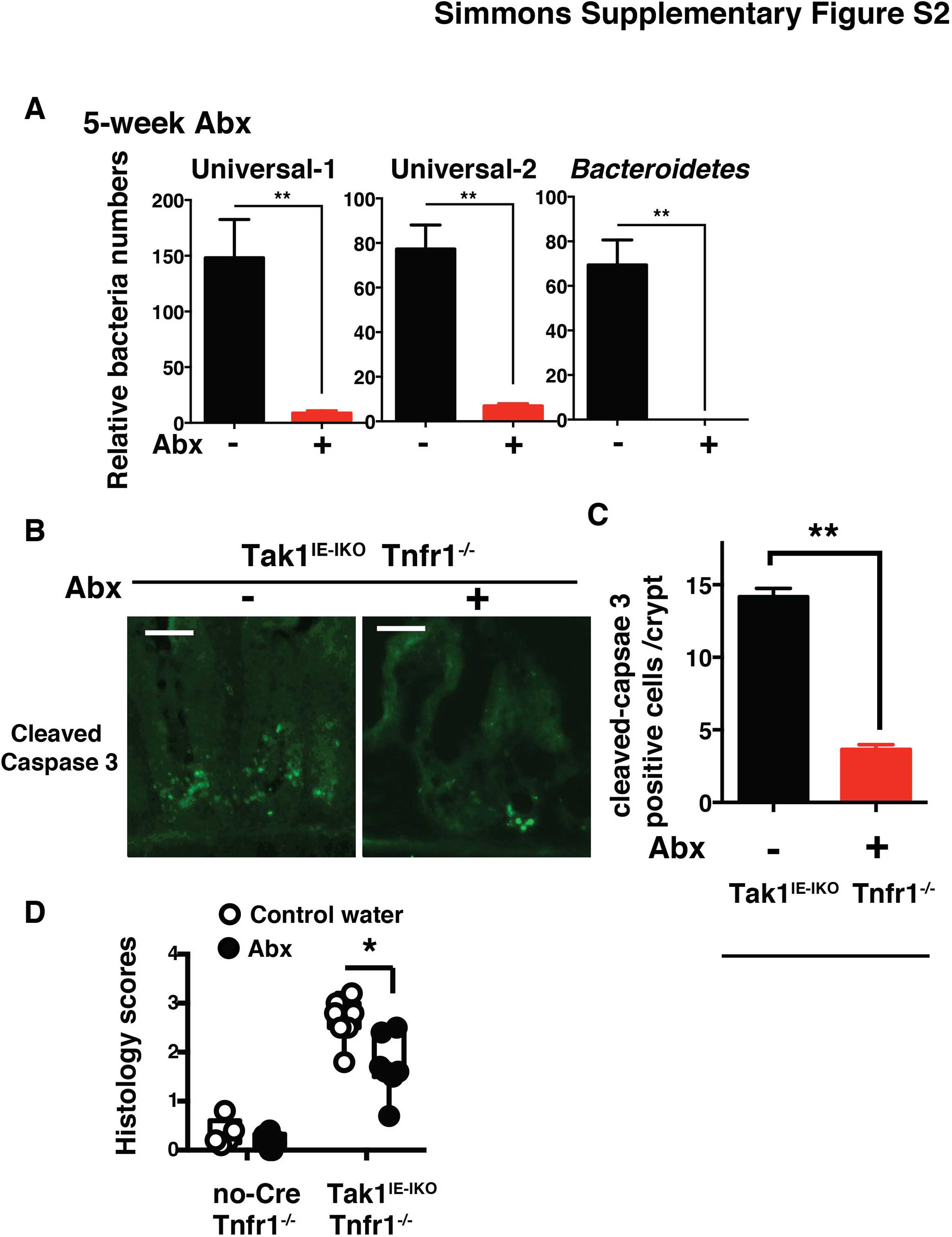

Supplement: Supplementary Figure 2 [file cddis201698x3.tif]

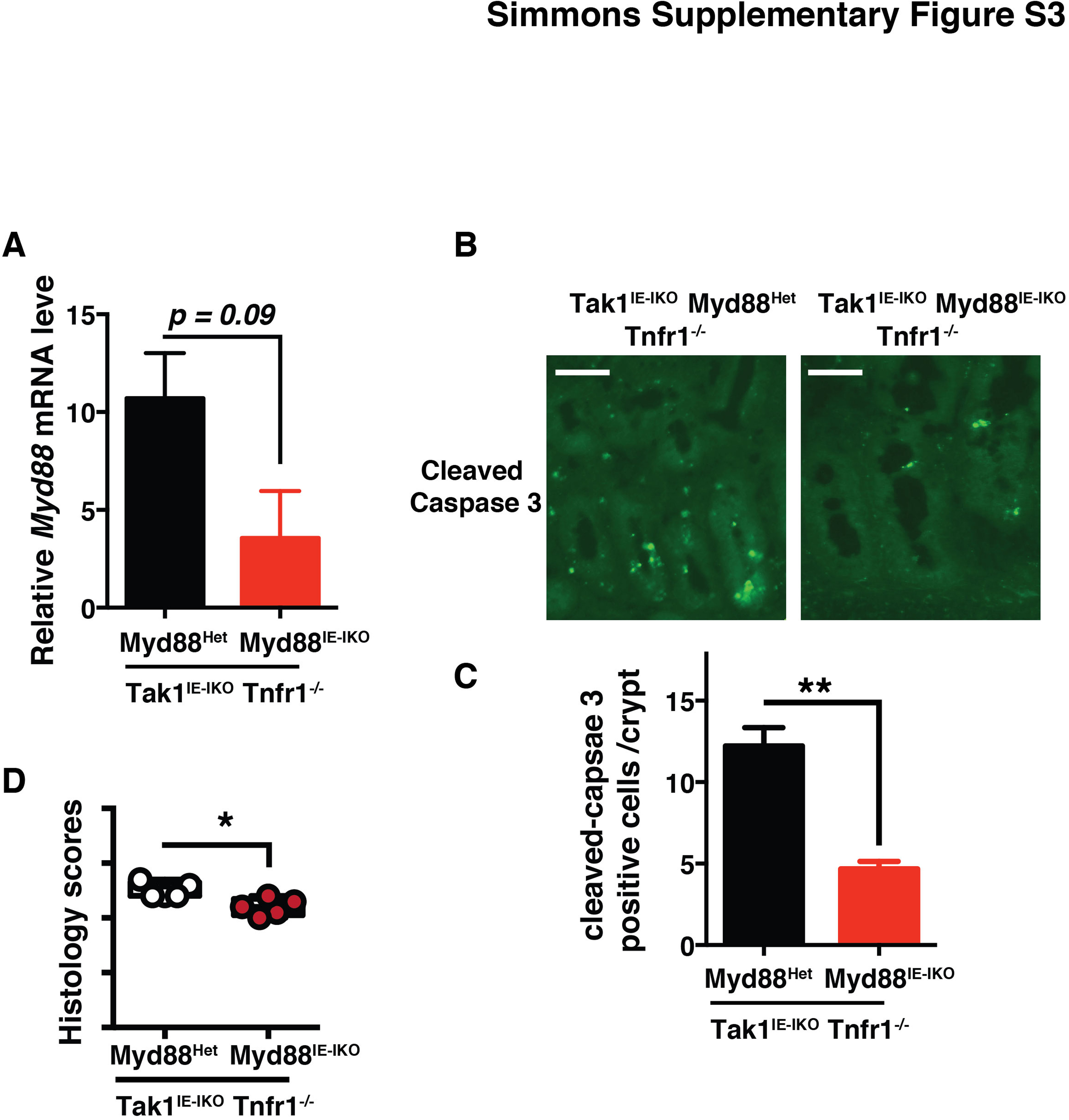

Supplement: Supplementary Figure 3 [file cddis201698x4.tif]

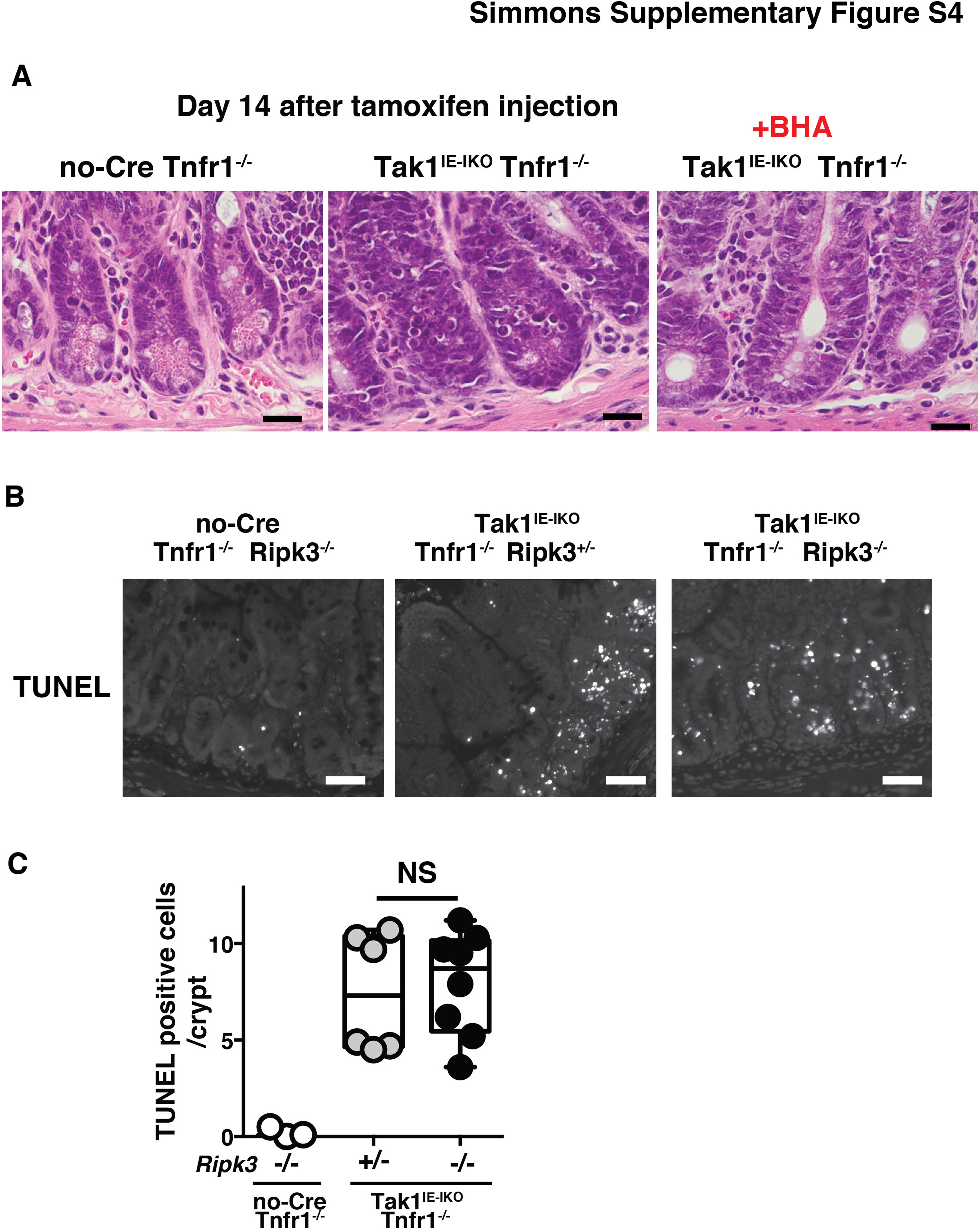

Supplement: Supplementary Figure 4 [file cddis201698x5.tif]
